# The Economic Impact of a Switch From Prescription-Only to Non-prescription Drugs in Italy

**DOI:** 10.3389/fphar.2018.01069

**Published:** 2018-10-17

**Authors:** Monica Hildegard Otto, Carla Pillarella, Claudio Jommi

**Affiliations:** ^1^Centre for Research on Health and Social Care Management (CERGAS) SDA Bocconi School of Management, Milan, Italy; ^2^Department of Social and Political Sciences, Bocconi University, Milan, Italy; ^3^Federchimica Assosalute, Milan, Italy; ^4^Department of Pharmaceutical Sciences, Università del Piemonte Orientale, Novara, Italy

**Keywords:** switch, OTC, economic impact, self medication, pharmaceutical expenditure

## Abstract

The paper analyses the potential economic impact of switching drugs from prescription-only to over the counter status, using Italy as a case-study. The study assumes a societal perspective, investigating the effects of switches (and consequent delisting) on drugs spending, avoided visits by GPs (General Practitioners) and avoided time spent by patients for these visits. It overcomes the main pitfalls of previous studies, providing a rational for listing switchable products and applying both a static (no impact of switch on prices and volumes consumed) and a dynamic approach (impact on pricing policies and volumes depending on price-elasticity). Different scenarios were assumed including shorter/longer time spent for visits and inclusion/exclusion of the economic value of time of retiree patients. Results show that switching policy provides with societal savings ranging from 1 to 2.1 1 billion Euro. The economic impact on patients is less straightforward and depends on the scenarios used. If a longer time is spent on visits, the economic value of this time will compensate the cost of the switch to patients due to delisting. Despite the net economic benefit should be carefully interpreted, the results demonstrate how switching can contribute to the sustainability of the health care system in the middle-long run thanks to the more rational use of resources, combined with an increased awareness and responsibility of the involved stakeholders.

## Introduction

The value of the regulatory switch from prescription (Rx) to non-prescriptionmedicines is a topic discussed in both scientific and gray literature and analyzed in different perspectives. Some contributions evaluate the effects of the regulatory switch (and, hence, the greater availability of non-prescription drugs) in terms of drug efficacy and patient management. For instance, the literature discusses how the increased access to non-prescription drugs, compared to Rx drugs, can foster greater adherence to therapy, resulting in the more effective prevention or treatment of minor pathologies (Brass et al., 2011). At the same time, the risks associated with the misuse of non-prescription drugs are taken into consideration, as well as the required path of education and training for the patient. The literature takes also into consideration the implications of a regulatory switch in terms of patient management, from the general practitioner (GP) perspective and the changing (i.e., more active) role of pharmacists(Andrade et al., 1999; Vamvakopoulos et al., 2008).

From an economic point of view, the literature analyzes the effects in terms of the following factors: (i) variation of the financial mix of pharmaceutical spending (the regulatory switch normally involves the delisting of a previously reimbursed drug); (ii) costs avoided by the payer due to the decrease in the number of visits to the GP; (iii) costs saved by citizens, such as avoided productivity loss.

Some studies estimate the potential impact of the switch on specific therapeutic classes, such as antihistamines, cough and cold preparations, and drugs for acid-related disorders (Temin, 1983, 1992; Sullivan et al., 2005; Ravis and Eaglstein, 2007). Other authors estimate a more general economic impact of switching drugs from Rx to non-prescription for some therapeutic classes in countries, such as Greece^1^, Spain^2^ and France (European Commission., 2015).

In general, these studies (i) present some approximation in the simulations (e.g., lack of a specific rationale for choosing the switchable molecules/packets, simplifying hypotheses on the number of avoided visits) and (ii) do not perform a sensitivity analysis that simulate show the variations of the most relevant parameters (unit prices and volumes) may affect the expenditure.

This paper aims at covering the literature gaps in estimating the economic impact of the regulatory switch, using Italy as a case study.

### The Italian pharmaceutical system

The Italian pharmaceutical system divides drugs into three categories: Class A, which includes pharmaceuticals for severe and chronic diseases reimbursed by the SSN (Servizio Sanitario Nazionale) in all settings; Class H, which includes drugs reimbursed only if used in hospital setting; and non-reimbursable drugs, which includes both some Rx and non-prescription drugs.

Reimbursement and ex-factory prices are simultaneously negotiated by the Italian regulatory Agency (AIFA) and the relevant company. The main criteria used during negotiation are the burden of disease, the place in therapy and the availability of alternative treatments, the risk-benefit profile, the therapeutic added value, and the impact on the drug budget.

For reimbursable drugs, distribution margins are regulated by law. At present (Law 122/2010), wholesalers and pharmacists receive a 3% and a 30.35% margin, respectively, on the final price before the value-added tax (VAT) of 10%. The distribution margin for generics is 8% higher (and margins for the industry are 8% lower) than for other drugs. If the drug is covered by the SSN, pharmacists are subject to a progressive discount ranging from 3.75% (when the drug's final price is under 25.82 Euros) to 19% (if the price is over 154.94); hence, actual margins for pharmacists range from 26.6 to 11.35%.

For non-reimbursable Rx drugs, the industry is free to set prices, but prices can be raised in each odd-numbered year. Distribution margins over list prices are free but usually aligned with the margins for drugs covered by the SSN.

For non-prescription drugs, both prices and distribution margins are free. Whereas, the final price of non-reimbursable Rx drugs is the same all over the country, prices for non-prescription drugs may differ across pharmacies and other points of delivery (para-pharmacies and gross retailers. Since 2006 (Law Decree 223/2006), para-pharmacies (3,156 in 2014) and gross retailers (340 areas in gross retailers) are authorized to distribute non-prescription drugs, provided that the sales are supervised by a pharmacist and that a separate area for drug sales is created (corner). In 2016, mass-retailer market shares were still very limited: 91% of the total market was sold in pharmacies, 5.2% was sold in para-pharmacies and only 3.5% was sold in mass retailers (Jommi and Minghetti, 2015).

From a regulatory standpoint, non-prescription drugs are out-of-pocket expenses, apart from exemptions for some people suffering from rare diseases. In the past they were distinguished between those that can be advertised to the general public and can be dispensed Over the Counter (OTC) and those that cannot (SOP). However, according to a recent judgment (Sentenza Consiglio di Stato 2217/2017) all non-prescription drugs can be advertised: hence, OTC and other non-prescription drugs are different only for their over and behind the counter nature, respectively.

Among European countries over the last 15 years (2001–2016) Italy has recorded one of the lowest growth rates for the overall (retail) pharmaceutical market (+0.1% on average) and the lowest growth rate in Rx drug expenditure (−0.1%; Assosalute, 2017). This trend is mainly a result of cost-containment policies (e.g., pharmaceutical spending caps, reference pricing, etc.) and patent expirations. On average, at the European level, the proportion of non-prescription drug expenditure on the total retail pharmaceutical expenditure represented in 2016 the 15.3% and Italy is among the countries with the lowest proportion (i.e., 13.9%). If compared with the European average per capita expenditure for OTC (47.9 euro in 2016), Italy is one of the countries with the lowest expenditure (29.7; Assosalute, 2017).

Starting from the comparison of the non-prescription behavior in the main European countries, this paper aims at analyzing the potential economic impact of switching drugs from Rx (either classified as A or as non-reimbursable) or from SOP to OTC. The authors have already studied this aspect, but they previously considered only the effects of the switch on pharmaceutical expenses (Jommi and Otto, 2013). The goal of this paper is to integrate this previous analysis, (i) with a broader (societal) viewpoint, i.e., including health care payers, other third-party payers and the citizens' perspectives and (ii) overcoming literature gaps, i.e., providing a rationale for switching choices, a more structured evaluation of avoided GPs visits and dynamic simulation.

## Materials and methods

This paper addresses five key methodological aspects. First, the products (molecules/packs) potentially subject to the regulatory switch (i.e., *switchable products*) must be identified. The potential switch was assessed on the grounds of the current classification^3^ of drugs in the major European markets, namely, France, Great Britain, Germany, and Spain.

European countries can autonomously take decisions concerning the switch of drugs from the prescription only to the non-prescription status. Although the European legislation applies to the switch, the final decision on the legal status remains a national prerogative^4^. Among the benchmark countries taken into consideration in this study (i.e., France, Great Britain, Germany, and Spain), Great Britain and Germany^5^have a long tradition of switch, while France is among the most conservative countries. In general, there are significant differences among European countries in terms of national switch requirements and processes.

To identify the switchable products (Table 1), three selection criteria were combined: (i) the product had to be classified in Italy as Rx or SOP, (ii) the products had to be classified as an OTC drug in at least one of the main European countries (France, UK, Germany, Spain), (iii) injectable packs were excluded, because they are not suitable for OTC status. With reference to Rx drugs, a reclassification to OTC was assumed, by assigning three switch priority levels: (i) high switch priority to products that were classified as OTC in at least three of the abovementioned countries; (ii) medium priority to products classified as OTC in two of the aforementioned countries; (iii) low priority to products classified as OTC in one of the above countries. With reference to the products in the SOP class, the reclassification hypothesis from SOP to OTC was applied with a homogeneous (high) degree of priority.

**Table 1:** List of switchable products and priority levels.

**Active ingredients**	**From A**			**From C (Rx)**			**From SOP**
	**High**	**Medium**	**Low**	**High**	**Medium**	**Low**	
Acetylcysteine				x			x
Almotriptan			x				
Azelastine				x			x
Azithromycin			x			x	
Benzydamine							x
Benzylbenzoate (topical)				x			
Budesonide (nasal)			x				
Chlorpheniramine				x			
Cinchocaine				x			x
Codeine		x			x		
Cromoglicid acid				x			x
Cyproheptadine				x			
Diosmin					x		x
Ebastine			x			x	
Econazole				x			x
Emedastine							x
Epinastine							x
Erdosteine						x	
Famotidine	x						
Fenticonazole				x			x
Flavoxate hydrochloride					x		
Fluconazole			x			x	x
Flunisolide (nasal)			x			x	
Fluticasone		x			x		
Fluticasone furoate		x		x			
Folic acid	x			x			x
Glucosamine				x			
Hydroxyzine						x	
Hymecromone							x
Indometacin			x			x	
Iron ferric	x			x			
Iron ferrous	x			x			
Iron metal				x			x
Iron succinyl-protein complex				x			
Isoconazole				x			x
Ketoprofen			x			x	
Ketotifen							x
Levonorgestrel			x	x			
Lidocaine	x			x			x
Loratadine	x			x			x
Macrogol(s)				x			x
Mebendazole			x				
Mebeverine						x	
Metronidazole							x
Miconazole	x			x			x
Minoxidil				x			x
Nitro-glycerine		x			x		
Nizatidine			x				
Oxatomide							x
PolymyxIn B						x	x
Prilocaine					x		
Promethazine	x			x			
Propantheline				x			
Pyrantel	x						
Rabeprazole			x				
Racecadotril				x			
Silver				x			x
Simvastatin			x				
Sucralfate			x				
Sulfacetamide							x

*Source: own elaboration on 2016 AESGP data*.

The second step of the analysis is to simulate the effects of the switch on the pharmaceutical expenditures in the absence of price variation (and consumption) for switched medicines (static approach). Starting from the 2015 national data on expenditure and volumes, detailed in terms of active ingredients, single package, and therapeutic class, the impact of a switch from both the payers' and citizens' perspectives was estimated. For drugs classified as non-reimbursable Rx drugs and SOP, there is no variation in payers because the products are not reimbursable either before or after the switch. Reimbursable drugs (A class) are partially covered by the SSN (70.4% of gross expenditure), citizens (co-payment, which accounts for 12.4% of gross expenditures; private expenditure on reimbursable products comprise 11.6% of gross expenditures) and the community pharmacies (discount on list prices account for 5.6%, on average; Pharmaceutical Observatory, 2016). After the switch, the full gross expenditure is covered by citizens.

The third methodological aspect is the estimation of the number of avoided visits and, through the unit cost per visit, the effects of the switch on avoided costs for GP visits (opportunity cost of the GP). The estimation of avoided GP visits is based on data related to (i) the number of packs for potentially switch products, (ii) the prescriptions issued by the SSN, (iii) the annual average number of visits to the GP per patient, weighted for the proportion of repeatable recipe (the prescription of a repeatable receipt does not require a visit).

The fourth step of the analysis estimates the economic impact of the switch on the time gained by citizens per avoided GP visit. To estimate the effects on the workers' loss of productivity during the visit, it was assumed that each patient could devote between 0.25 and 0.5 days per visit. This range, in the patients' perspective, includes the duration of the visit, but also the time spent to go to the GP, as well as the time lost in queuing and to return at work/home. This hypothesis, combined with the average income of employees (for the working-age population) and pensions (for the opportunity cost of time spent in visits), allows for the estimation of the economic value, from the perspective of the citizen, of the “lost” time for the visit to the GP. Since most of the economic analysis adopting the societal perspective include only workers' time lost, we estimated savings in two perspectives: the workers' perspective only and the workers and retired perspective combined.

In the end, the effects of the switch on pharmaceutical spending are estimated by introducing the hypotheses on price and consumption variations for drugs that lost the Rx status. From A to OTC, prices are supposed to increase from 10 to 30%. To estimate the impact of price increases on volumes, the price elasticity of demand for OTC medicines in 2015 (= −0.00378^6^) was applied. In other words, the positive change of 1% of the prices led to a decrease in consumption of 0.378%. The demand is not elastic enough to compensate for the change in price. In other words, a price increase is considered an expansive expenditure policy. In the case of switching from Rx non-reimbursable and SOP to OTC, a consumption growth up to 10 and 30%, respectively, is assumed. The reasons of this expected increase are (i) the increased visibility of the product, due to advertisement; (ii) (for drugs switch from Rx to OTC) the larger access, since OTC are available also outside community pharmacies.

Combining the effects of price and consumption variations on the pharmaceutical expenditure with those on avoided visits, three distinct scenarios can be formulated:

- a *static scenario* in which the effects on visits are associated only with the reallocation of spending due to the potential switches. This scenario is the result of the first area of analysis and does not incorporate the effects on prices and consumption;
- an *intermediate dynamic scenario* that, based on the static scenario, evaluates the effect of a small variation in both prices (of drugs reclassified from A to OTC, i.e., +10%) and consumption (from non-reimbursable and SOP to OTC, i.e., +10%);
- a *comprehensive scenario* that, starting from the basic scenario, introduces the effect of the maximum increase in the prices and consumption of drugs (+30% in the price of drugs reclassified from A to OTC, an increase in the consumption of drugs reclassified from non-reimbursable and SOP to OTC, 10 and 30%, respectively).

## Results

Results are reported for the whole switchable products (irrespective of switches priority)^7^ and the three above-mentioned scenarios (static, intermediate, and comprehensive scenarios). The economic value of time spent in visits was calculated using the 0.25 and 0.5 days lost hypotheses; time spent by retirees was estimated separately from working people and we provided two estimates, i.e., including time lost by both retirees and working people (opportunity cost of time spent in visits) and including time lost by working people only (productivity loss).

**Figure 1:**
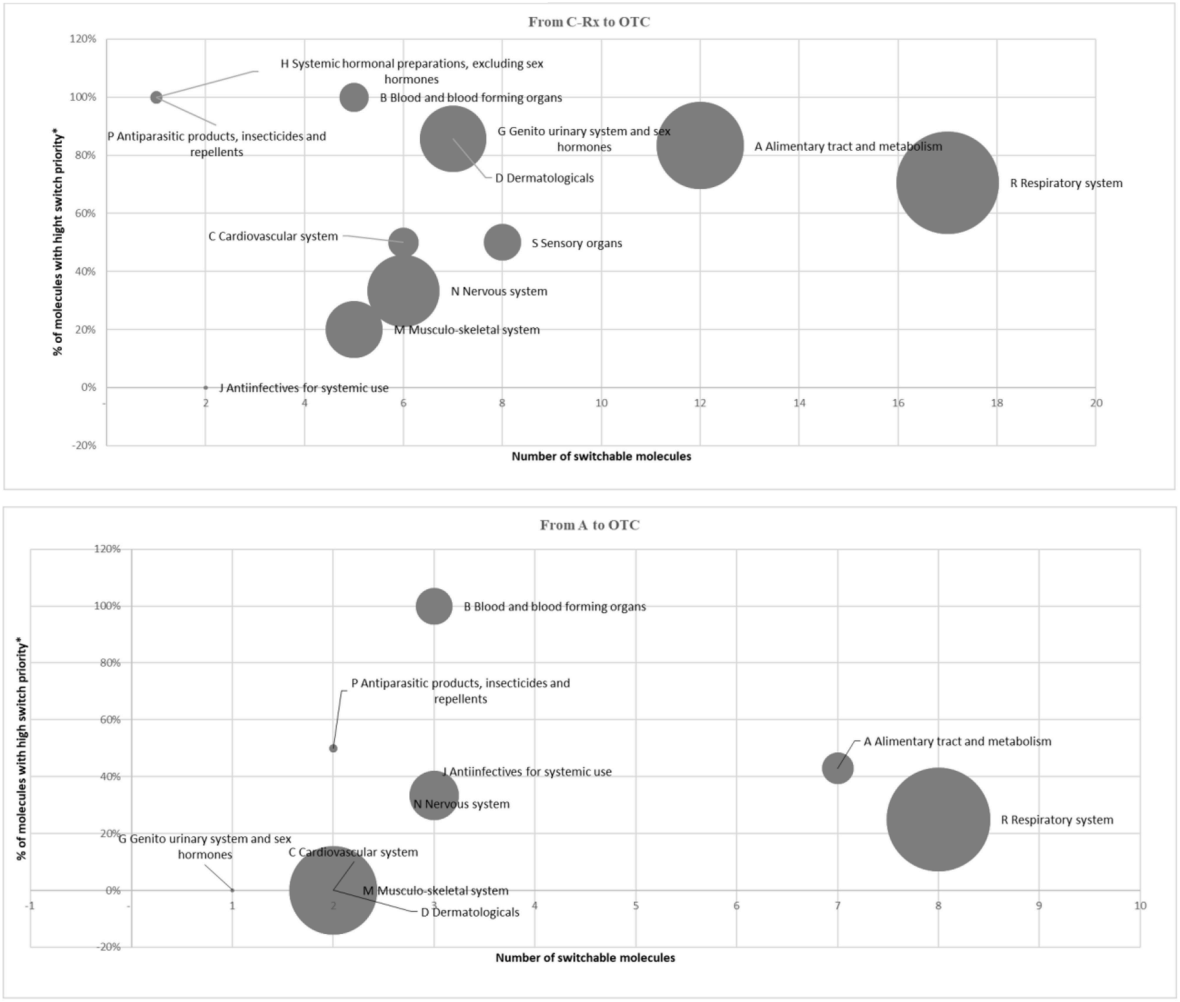
Market of switchable drugs by priority (2015). *Classified as OTC at least in 3 out of 4 main European Countries.

Following the described framework of analysis, in 2015, the potentially reclassified market can be calculated in 133.6 million packs, equivalent to ~8% of the total retail market of Italian drugs, for a value (in list prices) of 1.7 billion Euro, equivalent to ~10.5% of the total retail market of Italian drugs (Table 2). Figure 1 illustrates, for the Rx (reimbursable, i.e., A, and non-reimbursable, i.e., C) switch to OTC, the number of the involved molecules (abscissa axis), the incidence of high-priority switchable products (axis of the ordinates), and the size of the relevant market in terms of expenditure (bubble size). They demonstrate the following: (i) for the reimbursable drugs, the most important anatomical categories in terms of market size have a low switch priority (e.g., the cardiovascular and respiratory system drugs); (ii) for the non-reimbursable drugs, all major anatomical categories have a medium-high switch priority.

**Table 2:** Market of switchable drugs (2015).

**Items**	**Switchable market**	**Overall market 2015**	**%**
**Volumes (thousands of units)**	**133,567**	**1,662,800**	**8.0%**
Reimbursed prescription only (A)	95,965	1,336,523	7.2%
Non-reimbursed prescription only (C)	24,529	251,482	9.8%
Non-prescription (C)	13,073	74,795	17.5%
**Expenditure (million Euro)**	**1,677**	**15,979**	**10.5%**
Reimbursed prescription only (A)	1,198	12,295	9.7%
Non-reimbursed prescription only (C)	316	3,038	10.4%
Non-prescription (C)	162	646	25.1%

*Source: own elaboration on IQVIA data*.

Considering the size of the market under investigation and the proposed switching potential^8^, 34.2 million GP visits could be avoided (as detailed in Table 3). This decrease would lead, on the one hand, to an SSN opportunity cost (equal to 706 million Euro), and on the other hand, to a gained economic value for employees and retirees' time corresponding to 1.465 million Euro (1.119 and 274 million Euro, respectively).

**Table 3:** The economic value of avoided GP visits.

**Calculation**	**Item**	**Amount**	**Year**	**Source/comments**
1	Packs for reimbursed drugs (millions)	1,133.0	2015	Osmed, 2016
1*bis*	Packs for reimbursable drugs privately purchased (millions)	225.0		
1*ter*	Packs for C class drugs (millions)	248.0		
2	Prescriptions for reimbursed drugs (millions)	596.0		
2*bis* = 1*bis*/3	Prescriptions for private purchase (millions)	118.4		Packs not covered by the SSN have the same proportion of recipes prescriptions and packs referring to the consumption of SSN
2*ter* = 1*ter*/3	Prescriptions for C class (millions)	130.5		
3 = 1/2	Packs per prescription	1.90		
4 = switchablepacks/3	Avoided prescriptions (millions)	63.4		Switchable packs as presented in Table 2
5	Average yearly GP visits per patient	9.6	2014	Health Search, 2016
6	Over 14 population	52,383,692	01-Jan-2016	Istat (www.istat.it; accessed November 2016)
7 = 5*6	Yearly GP visits	502,883,443		
8 = 7/1	Prescriptions per GP visit	1.68		
**9** = ((4/8)*0,94*0,9) + ((4/8)*(0,06))	**Avoided GP visits (millions)**	**34.2**		Based on the national drug formulary, the 94% of the molecules potentially switchable are subject to repeatable recipe (RR). The hypothesis of a lower access (estimated −10%) to GP has been applied.
**9*****bis***	**Economic value of avoided GP visits (million Euro)**	**706.2**		Literature provides estimations of the economic value per avoided visit: 11,26 Euro Lucioni et al., 2005 and 12 Euro Garattini et al., 2003. Taking into account the inflation rate, the 2015 value is 20,66 Euro (that corresponds to the fee for specialist visits in the national tariff system—i.e., Nomenclatore Nazionale Tariffario)
10	15–65 population	39,740,275	01-Jan-2016	Istat (www.istat.it; accessed November 2016)
11 = 10/6	15–65 population (%)	76%		
12	Over 65 population	12,643,417	01-Jan-2016	Istat (www.istat.it; accessed November 2016)
13 = 12/6	Over 65 population (%)	24%		
14	Patients' days per visit	0.25–0.5		Own hypothesis
15	Average net income for employees (Euro)	33,516	2014	Istat (www.istat.it; accessed July 2017)
16 = 15/365	Daily average net income for employees (Euro)	92		
17 = 9*11*14*16	Economic value of employees' time (million Euro)	595.33–1,190.65		
18	Average net income for retirees	24,257	2014	Istat (www.istat.it; accessed July 2017)
19 = 18/365	Daily average net income for retirees	66		
20 = 9*13*14*19	Economic value of retirees' time (million Euro)	137.08–274.16		
**21** = **17** + **20**	**Economic value of employee** + **retiree time (million Euro)**	**732.41–1,464.81**		

The combination of the effects of avoided visits on the pharmaceutical expenditure is detailed in the static scenario (no increase in price and consumption). This analysis shows a reallocation of the drug expenditure from SSN to the citizens (Table 4). On the one hand, SSN saves 844 million Euro, generated—almost for the 50%—by middle-high priority switches among which the respiratory system treatments (mainly for asthma and COPD) and—with a lower impact—molecules for blood and blood forming organs (such as folic acid). The remaining 50% consists of low priority switches mainly represented by treatments for the cardiovascular system (such as statins). On the other hand, the private drugs expenditure (equal to 916 million Euro) corresponds to SSN savings, plus the value of the discount that the SSN benefited and the one applied in the case of private purchases of reimbursable medicines that are removed after the switch (see section Materials and Methods).

**Table 4:** The economic impact of switches.

**Item**	**Static scenario**			**Intermediate dynamic scenario**			**Comprehensive scenario**		
	**SSN (a)**	**Citizen/otherpayer (b)**	**Society (a + b)**	**SSN (a)**	**Citizen/otherpayer (b)**	**Society (a + b)**	**SSN (a)**	**Citizen/otherpayer (b)**	**Society (a + b)**
**MILLION EURO**									
Variation of drugexpenditure	−843.76	916.41	72.65	−843.76	961.63	117.86	−843.76	1,106.93	263.17
Avoided visits (SSN opportunity cost)	−706.24	–	−706.24	−706.24	–	−706.24	−706.24	–	−706.24
Avoided visits (economic value of employee time) (min)	–	595.33	595.33	–	595.33	595.33	–	595.33	595.33
Avoided visits (economic value of employee time) (max)	–	1,190.65	1,190.65	–	1,190.65	1,190.65	–	1,190.65	1,190.65
Avoided visits (economic value of retiree time) (min)	–	137.08	137.08	–	137.08	137.08	–	137.08	137.08
Avoided visits (economic value of retiree time) (max)	–	274.16	274.16	–	274.16	274.16	–	274.16	274.16
**Total (min)**	−**1,550.00**	**184.00**	−**1,366.00**	−**1,550.00**	**229.22**	−**1,320.79**	−**1,550.00**	**374.52**	−**1,175.48**
**Total (max)**	−**1,550.00**	−**548.40**	−**2,098.40**	−**1,550.00**	−**503.18**	−**2,053.19**	−**1,550.00**	−**357.88**	−**1,907.88**
**Total (only economic value of employee time) (min)**	−**1,550**	**321.08**	−**1,228.92**	−**1,550.00**	**366.30**	−**1,183.71**	−**1,550.00**	**511.60**	−**1,038.40**
**Total (only economic value of employee time) (max)**	−**1,550**	−**274.24**	−**1,824.24**	−**1,550.00**	−**229.02**	−**1,779.03**	−**1,550.00**	−**83.72**	−**1,633.72**
**PERCAPITA (EURO)**									
Variation of drugexpenditure	−16.11	17.49	1.39	−16.11	18.36	2.25	−16.11	21.13	5.02
Avoided visits (SSN opportunity cost)	−13.48	–	−13.48	−13.48	–	−13.48	−13.48	–	−13.48
Avoided visits (economic value of employee time) (min)	–	11.36	11.36	–	11.36	11.36	–	11.36	11.36
Avoided visits (economic value of employee time) (max)	–	22.73	22.73	–	22.73	22.73	–	22.73	22.73
Avoided visits (economic value of retiree time) (min)	–	2.62	2.62	–	2.62	2.62	–	2.62	2.62
Avoided visits (economic value of retiree time) (max)	–	5.23	5.23	–	5.23	5.23	–	5.23	5.23
**Total (min)**	−**29.59**	**3.51**	−**26.08**	−**29.59**	**4.38**	−**25.21**	−**29.59**	**7.15**	−**22.44**
**Total (max)**	−**29.59**	−**10.47**	−**40.06**	−**29.59**	−**9.60**	−**39.19**	−**29.59**	−**6.83**	−**36.42**
**Total (only economic value of employee time) (min)**	−**29.59**	**6.13**	−**23.46**	−**29.59**	**7.00**	−**22.59**	−**29.59**	**9.77**	−**19.82**
**Total (only economic value of employee time) (max)**	−**29.59**	−**5.24**	−**34.83**	−**29.59**	−**4.37**	−**33.96**	−**29.59**	−**1.60**	−**31.19**

SSN and citizens then benefit from a lower cost generated by avoided visits, in terms of (i) opportunity cost of time dedicated to avoided visits for the SSN; productivity gains (for workers); opportunity cost of time spent in visits (for retired people). Therefore, overall, in the static scenario, after the proposed switch, the lower costs in the social perspective amount between ~1.4 (in case of *minimum patients' days per visit*, i.e., 0.25) and 2.1 (in case of *maximum patients' days per visit*, i.e., 0.5) billion Euro (respectively corresponding to 26 and 40 Euro per capita). If the economic value of retirees' time would not be considered, the lower costs in the social perspective would range between 1.2 and 1.8 billion Euro (respectively corresponding to 23 and 35 Euro per capita).

The intermediate dynamic scenario results in a cost reduction between ~1.3 (in case of *minimum patients' days per visit*, i.e., 0.25 days) and 2.05 (in case of *maximum patients' days per visit*, i.e., 0.5 days) billion Euro (respectively corresponding to 25 and 39 Euro per capita). The SSN is not affected by a variation in costs if compared with the static scenario, as the effects of price and consumption variations have an impact once the drugs are reclassified and are therefore no longer covered by the SSN. The out-of-pocket expenditure increases because of the increase in consumption for the drugs reclassified from Rx non-reimbursable and SOP and the rise in prices for the drugs reclassified from A (including the effect of the discounts on SSN and on the private purchase of reimbursable drugs, see above). This increase in prices more than compensates for the reduction in consumption due to the low price-elasticity of the demand. Without the economic value of retirees' time, the lower costs in the social perspective would range—as in the static scenario—between 1.2 and 1.8 billion Euro (respectively corresponding to 23 and 34 Euro per capita).

In the comprehensive scenario, applying the same logical path illustrated for the intermediate scenario, the cost reduction for society is expected to be between 1.2 (in case of *minimum patients' days per visit*, i.e., 0.25) and 1.9 billion Euro (in case of *maximum patients' days per visit*, i.e., 0.5). The economic value of lower costs in the social perspective respectively falls between 22 and 36 Euro per capita. If only productivity loss is included, fall in costs in the social perspective would range—as in the static scenario—between 1 and 1.6 billion Euro (respectively corresponding to 20 and 31 Euro per capita).

## Discussion and conclusions

Our analysis shows that the switch from Rx to non-prescription status of the selected drugs for minor diseases produces important savings in public spending for drugs (844 million Euro): 50% of these potential savings derive from medium-high priority switches. These savings may be allocated to new and cost-effective drugs for severe diseases and/or unmet needs, in a context of scarce resources. Delisting of reimbursable drugs following the switch would imply an increase in out-of-pocket payment ranging from 17.5 to 21 Euro per capita yearly. Despite this undoubtable increase, the per capita expenditure for OTC drugs would remain aligned with that of Europe: in 2016, Italian citizens spent an average 31 Euro on OTC drugs, compared to 47.9 Euro spent in Europe (Assosalute, 2017).

Another important effect for health care payers are the avoided visits for GPs. This does not imply any saving because GPs are paid on a per-capita and not a fee-for-service basis. However, the time saved from avoided visits for minor diseases may be re-allocated to more severe illnesses and more complex patients, with a potential increase in allocative efficiency.

From the social perspective, society may gain a reduction of costs, ranging from 1.4–2.1 billion Euro in the static scenario to 1–1.9 billion Euro, should there be the maximum expected rise in the switched drugs.

The positive economic impact would be even higher if we consider that enlarging the role of self-medication for minor diseases would make patients, in principle, more conscious of their needs and avoid inappropriate demand for drugs. We are aware that in the short run patients may incur in possible drugs misuse or abuse, but this could be faced with the pharmacists' vigilance and patients' education by GPs. Moreover, as suggested by the literature (Creyer et al., 2001), misuse can be prevented by the proper information to consumers. If the latter are aware of the capabilities of OTC drugs, the risks associated with taking these drugs and the circumstances under which a visit to the doctor is the right choice, then the patients who visit the doctor will do so only when they have symptoms or a condition that they feel unable to treat using OTC drugs.

The study has some limitations.

Even though our simulations are better grounded (rationale for switches, accurate estimates of avoided visits), they still rely on many hypotheses (e.g., 0.5/0.25 days lost for a visit).

Furthermore, we could not rely on drugs and disease-specific data. For example, it is likely that private expenditure on reimbursable drugs and the number of GPs' visits depends on many variables, including the drug price, and the disease severity, respectively. As for the former we preferred to avoid adjustments that could turn into less reliable estimates; as for the latter, we have incorporated the effects of the disease on the number of visits looking at the prevalence, among switchable drugs, of products which receipt can be repeated. For repeatable receipts, the number of visits per prescription was reduced.

In principle, the delisting, due to the switch from reimbursable to OTC status, might generate a shift from the switched product to other reimbursable drugs for the same target. This shift could partially compensate savings from switches. The interchangeability between prescription-only and OTC drugs for the same target has been already investigated (Jommi, 2018). However, the relevant data were not used since (i) they cover therapeutic classes not fitting with the ones we have considered in the present study; (ii) the potential shift from prescription-only to OTC drugs was investigated, and not vice versa.

Furthermore, as already stated, the net economic benefit is the result of impacts that are not perfectly comparable: some are financial impacts and others are economic impacts, with any financial consequences.

Finally, for the dynamic analysis, we could not rely on a complete database to estimate specific price-elasticity of the demand for drugs losing the reimbursement status. The dynamic analysis relies on an estimate of the aggregate price-elasticity of the demand for OTC drugs to a change in their prices. Since patients are often required a co-payment on reimbursable products and unit prices for switchable drugs are often quite low, but higher than co-payment, our estimates should be very similar to what we could have found from a complete database.

Despite these limitations, this research has overcome limitations of previous studies on the effects of regulatory switches and has provided robust estimates of these switches in the Italian context, showing their contribution to the sustainability of the health care system in the medium to long term due to re-allocation and a more rational use of resources combined with an increased awareness and responsibility of the involved stakeholders. We are aware that the risk of drugs abuse and/or misuse should be faced and that the political resistance may play against a policy that produces increased drugs costs to patients. However, finding out resources for innovative and cost-effective drugs requires a rational allocation or resources and switch policies mag go in this direction.

## Author contributions

All authors contributed to the conception and design of the study. All authors were involved in the interpretation of the results, and contributed to drafting and revising the manuscript. All authors approved the final version of the manuscript.

### Conflict of interest statement

The research was carried out thanks to an unconditional grant from Federchimica Assosalute. CP is an employee of Assosalute Federchimica. The remaining authors declare that the research was conducted in the absence of any commercial or financial relationships that could be construed as a potential conflict of interest.

## Footnotes

^1^Maniadakis N. The economic value of moving medicines from prescription to non- prescription status: Case Study Greece, 51st AESGP Annual Meeting, 26–28 May 2015.

^2^Pellisé L. and Serra M. The economic impact of a hypothetical Rx-To-OTC switch in Spain, 18th ISPOR Annual Meeting, 7–11 November 2015.

^3^As provided on the Association of the European Self-Medication Industry web site: http://www.aesgp.eu.

^4^www.topra.org, Regulatory Rapporteur – Vol 14, No 12, December 2017 (Accessed May, 2018).

^5^http://www.thieme-connect.com (Accessed May, 2018).

^6^Calculations from the CERGAS SDA Bocconi Pharmaceutical Observatory on Quintiles IMS (now IQVIA) data.

^7^Calculations for medium-high priority switches are available at the reader's request.

^8^The results here presented refer to the entire set of switchable products. The authors are available to provide the reader with the analysis detailed in terms of priority level.
