# Corrigendum: The Economic Impact of a Switch From Prescription-Only to Non-prescription Drugs in Italy

**DOI:** 10.3389/fphar.2019.00129

**Published:** 2019-03-05

**Authors:** Monica Hildegard Otto, Carla Pillarella, Claudio Jommi

**Affiliations:** ^1^Centre for Research on Health and Social Care Management (CERGAS) SDA Bocconi School of Management, Milan, Italy; ^2^Department of Social and Political Sciences, Bocconi University, Milan, Italy; ^3^Federchimica Assosalute, Milan, Italy; ^4^Department of Pharmaceutical Sciences, Università del Piemonte Orientale, Novara, Italy

**Keywords:** switch, OTC, economic impact, self-medication, pharmaceutical expenditure

In the original article, there was a mistake in Table 2 as published. The item “NON prescription (C and Cbis)”, should have been listed as “NON prescription (C)”. Additionally, there was a mistake in Figure 1 as published. The horizontal axis interval was incorrect, therefore rendering the labels ineligible. The corrected Table 2 and Figure 1 appears below.

**Table 2:** Market of switchable drugs (2015).

**Items**	**Switchable market**	**Overall market 2015**	**%**
**Volumes (thousands of units)**	**133,567**	**1,662,800**	**8.0%**
Reimbursed prescription only (A)	95,965	1,336,523	7.2%
NON Reimbursed prescription only (C)	24,529	251,482	9.8%
NON prescription (C)	13,073	74,795	17.5%
**Expenditure (million Euro)**	**1,677**	**15,979**	**10.5%**
Reimbursed prescription only (A)	1,198	12,295	9.7%
NON Reimbursed prescription only (C)	316	3,038	10.4%
NON prescription (C)	162	646	25.1%

*Source: own elaboration on IQVIA data*.

**Figure 1:**
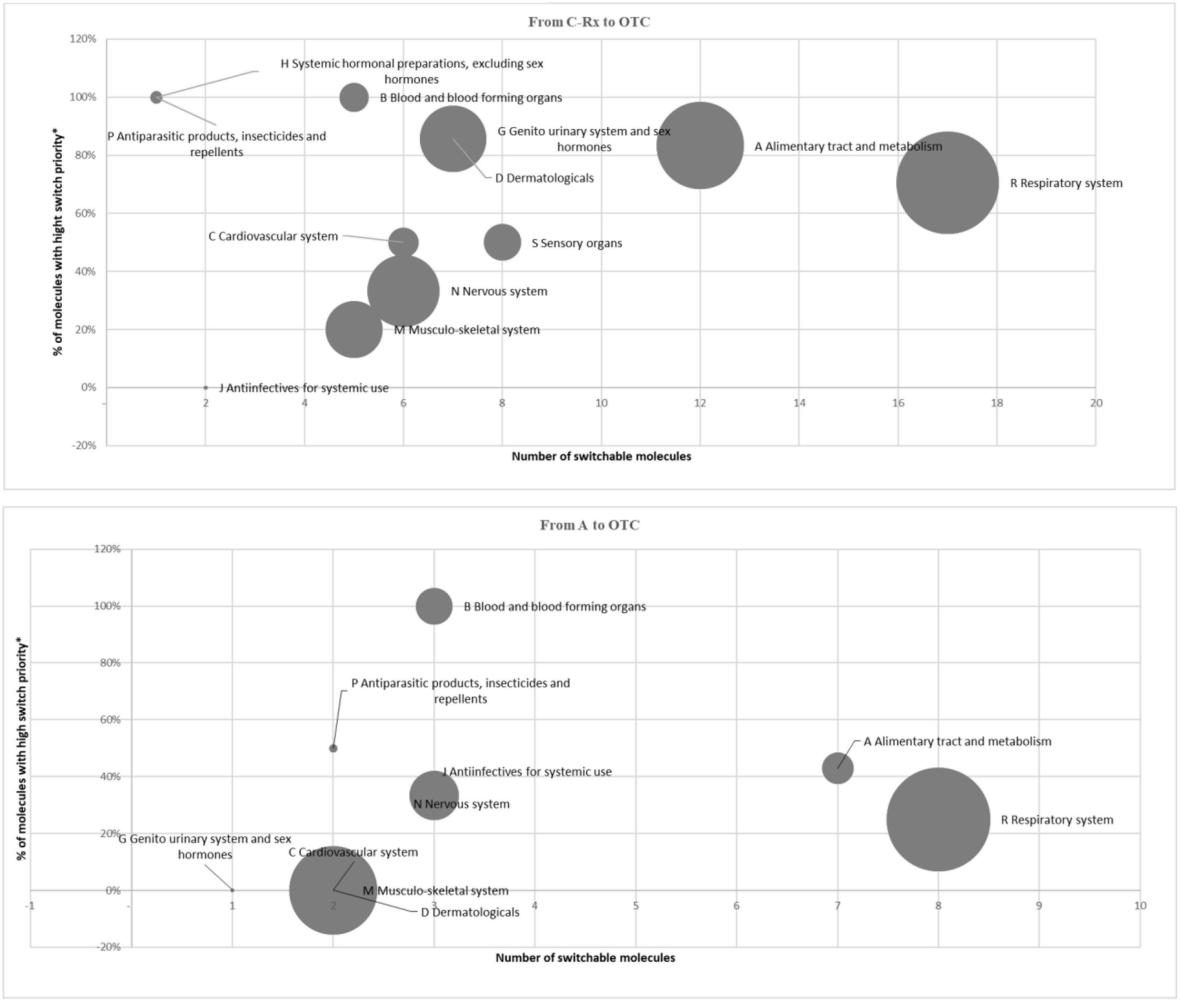
Market of switchable drugs by priority (2015). *Classified as OTC at least in 3 out of 4 main European Countries.

The authors apologize for this error and state that this does not change the scientific conclusions of the article in any way. The original article has been updated.

